# Research on Under Sleeper Pads for reducing vibrations in railway transport

**DOI:** 10.1038/s41598-026-56703-2

**Published:** 2026-06-06

**Authors:** Łukasz Chudyba, Wit Derkowski, Małgorzata Urbanek, Tomasz Lisowicz

**Affiliations:** Faculty of Civil Engineering, Cracow University of Technology, Kraków, Poland

**Keywords:** Railway track, Under Sleeper Pad (USP), Vibration isolation, Dynamic testing, Acceleration measurement, Track substructure, Elastic element, Engineering, Materials science

## Abstract

This study investigates the vibration isolation performance of under-sleeper pads (USPs). A reduced-scale laboratory track model was used, with simultaneous acceleration and displacement measurements at the sleeper substitute and at the subgrade surrogate. Five USP variants differing in stiffness and bottom-surface geometry were tested against a baseline configuration without a pad under harmonic excitation at 3, 5, 10 and 20 Hz (three repetitions per case). Vibration transmission was quantified using transfer functions expressed as the ratio of subgrade-to-block responses for RMS acceleration and peak-to-peak displacement. The results show a consistent trade-off: installing a USP increases the motion of the sleeper substitute (higher block RMS acceleration and displacement) due to added compliance, while reducing vibration levels at the subgrade surrogate. The attenuation strongly depends on pad stiffness and geometry, indicating that both material properties and surface design significantly influence vibration transmissibility. Softer and corrugated variants yielded the lowest transfer-function values and the highest vibration isolation. Based on the quantitative ranking, one optimized pad variant (characterized by unit static stiffness ≈ 0.12 N/mm³ and a corrugated contact surface) was selected for planned in-track verification on the PKP PLK network (Polish Railway Lines).

## Introduction

The application of engineering solutions aimed at protecting the surroundings of rail infrastructure from noise and vibrations has become increasingly prevalent. This trend has intensified in recent years. In case of newly designed tram or metro lines, such solutions are required already at the conceptual design stage. Conversely, in conventional railway transport — which predominantly operates outside densely populated urban areas — these solutions remain relatively uncommon in many countries, for example in Poland.

In Europe, this issue has already been recognised by legislators. This is reflected in the introduction of harmonised legal frameworks and technical guidelines addressing both urban environments and less densely populated areas. From a regulatory perspective, the European Union’s Directive 2002/49/EC on the assessment and management of environmental noise^1^ establishes a framework for evaluating and mapping noise exposure levels, while also requiring competent authorities to develop action plans for noise reduction. Furthermore, vibration impact assessments for rail projects are often guided by standards such as ISO 14837^2^ (mechanical vibration — ground-borne noise and vibration from rail systems) and national equivalents. The implementation of new solutions in the track structures requires a series of tests and analyses of the impact on safety environment. The design process should be concluded with the installation of the solution on a test section — typically a research test track. As a preliminary step, laboratory tests are usually conducted to assess key performance parameters before field implementation^22^.

Laboratory testing of railway track structures is purpose-driven due to the system’s unique nature, considering the track as infinitely long. Individual components such as fastenings, sleepers, and ballast are tested in accordance with applicable standards. A key aspect is the scale effect — to accurately replicate the behavior of the track in laboratory conditions, an appropriate test stand must be developed. A comprehensive analysis of laboratory testing of railway track systems can be found in the work by Najafi et al.^19^. The authors emphasize that selecting the appropriate scale of laboratory testing and equipment configuration is crucial for reliable interpretation of results. Their findings indicate that proper scaling significantly improves the representativeness of data and supports more efficient infrastructure design decisions, particularly when supported by multicriteria decision-making methods such as TOPSIS (Technique for Order Preference by Similarity to Ideal Solution). The most important part of the research presented in the paper involves studying the mechanical behavior of track and railway infrastructure under various static and dynamic loads.

Previous research on railway infrastructure performance encompasses a broad range of analytical, numerical, and experimental approaches. Modeling-based studies support system-level optimization and operational planning^20^, while investigations of track components address structural durability and long-term mechanical performance^21^.

In the context of vibration mitigation, studies are typically divided into three main categories: numerical modeling of train–track interaction, field measurements on operational lines, and laboratory-scale experiments. Laboratory testing at reduced scale is particularly valuable because it enables controlled variation of parameters such as stiffness, boundary conditions, and excitation frequency, while ensuring repeatability and safety^23^.

Insufficient flexibility of the railway track, generating low deflection and increased dynamic forces from rolling stock, as well as higher traffic loads, lead to excessive degradation of the railway track, especially the ballast. These phenomena are amplified by repeated high-frequency dynamic loads, leading to a loss of uniformity of contact between the sleepers and the ballast and insufficient energy dissipation in the track structure. These phenomena result in higher noise and vibration levels for the surrounding area^1^.

Elastic Under Sleeper Pads (USPs) assembled on prestressed concrete sleepers are designed to increase the contact area with the ballast, thereby reducing surface deformation during operation. Additionally, they reduce vibrations propagated in the surroundings of the railway tracks^3,9^. These characteristics lead to a continuous increase in the use of such pads, including on high-speed lines. Publications^7,10–12^ provide examples of studies on the effectiveness of using Under Sleeper pads in terms of track position stability and durability. The findings from these studies confirm that USPs contribute significantly to reducing contact stresses between sleepers and ballast^7^, improving the long-term structural performance and extending maintenance intervals. Potocan^10^ highlights that the application of USPs enhances lifecycle cost efficiency, making them a sustainable solution for high-quality track construction. Sol-Sánchez et al.^11^ show that using recycled tire-derived materials as elastic pads improves lateral track resistance and mitigates degradation effects over time, demonstrating both environmental and structural benefits. Ngamkhuong and Kaeumnuon^12^ demonstrate through dynamic testing that USPs can effectively reduce the amplitude of impact loads on prestressed concrete sleepers, thereby lowering the risk of cracking and fatigue-related failures under high-intensity traffic conditions.

Due to the application of elastic Under Sleeper pads, the load is distributed among a higher number of sleepers, which extends the track deflection line and reduces pressure on the ballast. The use of high quality materials (especially polyurethanes) allows the Under Sleeper pads to ideally fit the concrete sleeper surface, without puncturing or damaging it during operation in track. In a newly built railway track, the contact surface between the sleeper and ballast is 2–8%. The use of USPs may increase the effective contact area to approximately 35% for pads with unit static stiffness around 0.2 N/mm³, resulting in a 10–25% reduction in ballast contact pressure^13^. Reduction of dynamic loads and the resulting vibrations translates into smaller displacement of ballast bed and track settlement^12^.

The long-term durability of prestressed concrete sleepers is known to be affected by cracking in the rail seat zone and material-related degradation mechanisms such as delayed ettringite formation (DEF)^24–26^. Since sleepers are designed for service lives of 40–50 years^14^, any bonded Under Sleeper Pad must ensure stable mechanical properties, particularly with respect to static and dynamic stiffness as well as fatigue resistance under repeated loading. Harmonised requirements and testing procedures for USPs, including bonding integrity and durability-related performance, are specified in EN 16,730^15^.

Depending on the manufacturer, panels are made of, among others, polyurethane foam, rubber granulate with polyurethane binder, polyethylene (EVA) or polyurethane composites. Panels are manufactured as multilayer elements with different assembling layer configurations on the surface of contact with concrete. Depending on the applied material and the USP type (i.e. on the manufacturer), the pads consist of e.g. assembling mesh, spring layer, and the alternative load deflection protective layer or of geotextile on one or both sides of the dampening element. Despite the extensive use of Under Sleeper Pads (USPs) in modern railway infrastructure, the quantitative assessment of their vibration isolation performance under controlled laboratory conditions remains limited. Existing studies have primarily focused on dynamic train–track interaction and vibration attenuation using Under Sleeper Pads^4,5^, sleeper–ballast interaction mechanisms and contact behavior^27^, ballast mechanical performance with and without USPs^28^, lifecycle and maintenance benefits of USPs^8^, or combined experimental and numerical investigations of static and dynamic track response under heavy-haul loading conditions^29^. While these studies provide valuable insight into track mechanics and material behavior, systematic laboratory-scale quantification of vibration transmissibility based on simultaneous acceleration and displacement measurements at both sleeper and subgrade levels has not been sufficiently addressed.

In particular, the trade-off between increased sleeper mobility and reduced vibration transmission to the substructure has not been sufficiently quantified using consistent experimental procedures. Furthermore, the combined influence of pad stiffness and bottom-surface geometry on vibration transmissibility in a layered ballast system requires further clarification.

Therefore, the objective of this study was to experimentally evaluate the vibration isolation performance of Under Sleeper Pads using a reduced-scale laboratory track model. The research aimed to:

- quantify acceleration- and displacement-based vibration transmissibility between sleeper and subgrade layers,
- investigate the influence of pad stiffness and bottom-surface geometry on isolation efficiency,
- identify the pad variant providing the most favorable balance between sleeper mobility and subgrade vibration reduction.

To achieve this objective, a structured experimental framework was developed, encompassing material characterization, system-level stiffness assessment, and dynamic excitation tests under controlled harmonic loading conditions. The results provide quantitative insight into the isolation mechanisms of USPs and support the selection of an optimized pad configuration for further in-track verification.

**Fig. 1:**
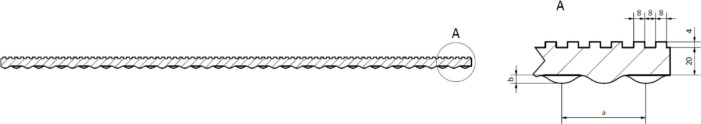
Geometry of new, developed USP. Own sources.

This solution has been submitted for protection at the Polish Patent Office^16^. Figure 1 shows the final geometry of the developed rail base pad.

## Materials and methods of Under Sleeper Pad testing

The tests were conducted at the Laboratory for Building Materials and Structures at the Cracow University of Technology, holding broad accreditation from the Polish Accreditation Centre (part of the European accreditation system) for testing railway track components.

The investigation was structured as a multi-stage experimental and analytical protocol. It aimed to determine the mechanical and vibration isolation properties of Under Sleeper Pads (USPs) under reduced-scale laboratory conditions.

Standardized testing procedures were not applied; instead, all tests were designed as custom experimental and developmental assessments tailored to the non-standard geometry and functional characteristics of the components under evaluation.

The experimental methodology comprised the following steps:

**Stage 1: Conceptual Framework and Experimental Model Development**.

- **Objective**: Define the physical modelling assumptions for a reduced-scale railway track system.
- **Description**: The experimental model retained the actual ballast thickness, while the remaining cross-sectional dimensions were proportionally scaled down. Concrete blocks were used in place of standard sleepers.
- **Rationale**: This configuration enables accurate replication of mechanical interactions between track components within the constraints of a laboratory-scale setup.

**Stage 2: Material Characterization – Determination of Fundamental Parameters**.

- **Objective**: Determine the static stiffness and material damping characteristics of actual samples, including:
  - Under Sleeper pads,
- **Scope**:
  - Testing was conducted for multiple material variants,
- **Rationale**: The obtained material properties form the foundation for subsequent system-level analyses, facilitating the assessment of material performance prior to integration with the track model.

**Stages 3–4: Theoretical Analyses and Variant Preselection**.

- **Objective**: Narrow the range of tested USP variants through theoretical analysis and preliminary modeling.
- **Description**: Analytical computations were performed to evaluate the influence of geometric and stiffness parameters on vibration transmission behavior.
- **Outcome**: Identification of the most promising variants for further experimental validation.

**Stage 5: Vibration Transfer Function Assessment – Track Model Evaluation**.

- **Objective**: Determine the vibration transfer functions of the reduced-scale track model under various configuration scenarios:
  - Baseline (no vibro-isolation components),
  - USP only,
- **Additional Analysis**: Measurement of system-level stiffness and damping for each integrated configuration.
- **Rationale**: This stage enabled a comprehensive evaluation of the vibro-isolation effectiveness of individual and combined solutions in a realistic yet controlled laboratory setting.

Subsequent in-track validation of the selected USP variant is planned and will be reported separately.

The tests were designed with the ultimate goal of installing single type of Under Sleeper Pad in the track. Due to the high costs of track testing, the research plan was structured to progressively reduce the number of designed solutions. Initially, five samples were tested on a small test site, then the number was reduced to three variants, and ultimately, one solution was selected for operational testing within the track.

## Laboratory tests

### Test of under sleeper pads

#### Test description

To design appropriate solutions for laboratory tests, the USPs were first tested to determine their physical properties — particularly stiffness-^17^. The samples were designed and manufactured by InnoEco, a project partner.

Table 1 Mechanical properties and geometry of tested Under Sleeper Pad variants.

**Table 1:** Type of tested Under Sleeper Pads (USP).

Tested USP			
USP type	USP static stiffness [kN/mm]	Combined block–USP static stiffness [kN/mm]	Shape
S1	3,80	13,95	Flat
S2	5,64	3,62	Sinusoidal (a=20 mm)
S3	3.90	4,32	Sinusoidal (a = 39,95 mm, density 0,8 kg/dm^3^)
S4	3.60	5,07	Sinusoidal (a = 39,95 mm, density 0,7 kg/dm^3^)
S5	3.4	4,32	Sinusoidal (a = 39,95 mm, density 0,6 kg/dm^3^)

The tested variants differed primarily in static stiffness and bottom-surface geometry, parameters expected to influence vibration transmissibility at the sleeper–ballast interface.

**Fig. 2:**
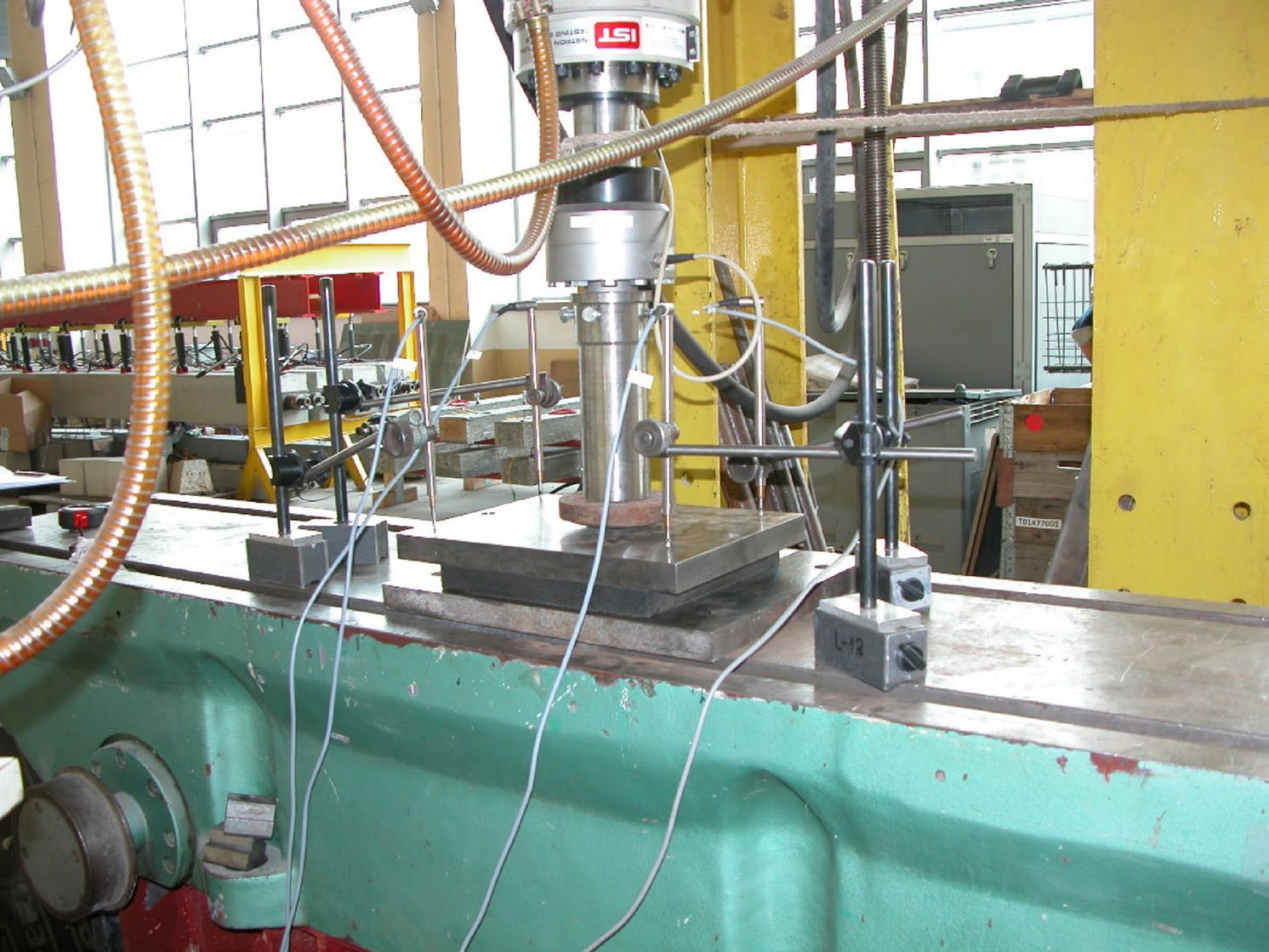
View of the setup for static and dynamic testing of material samples. Own sources.

The pad tests were performed using an Instron Schenck testing system equipped with digital measurement instrumentation from Hottinger Baldwin Messtechnik (Fig. 2).

**Fig. 3:**
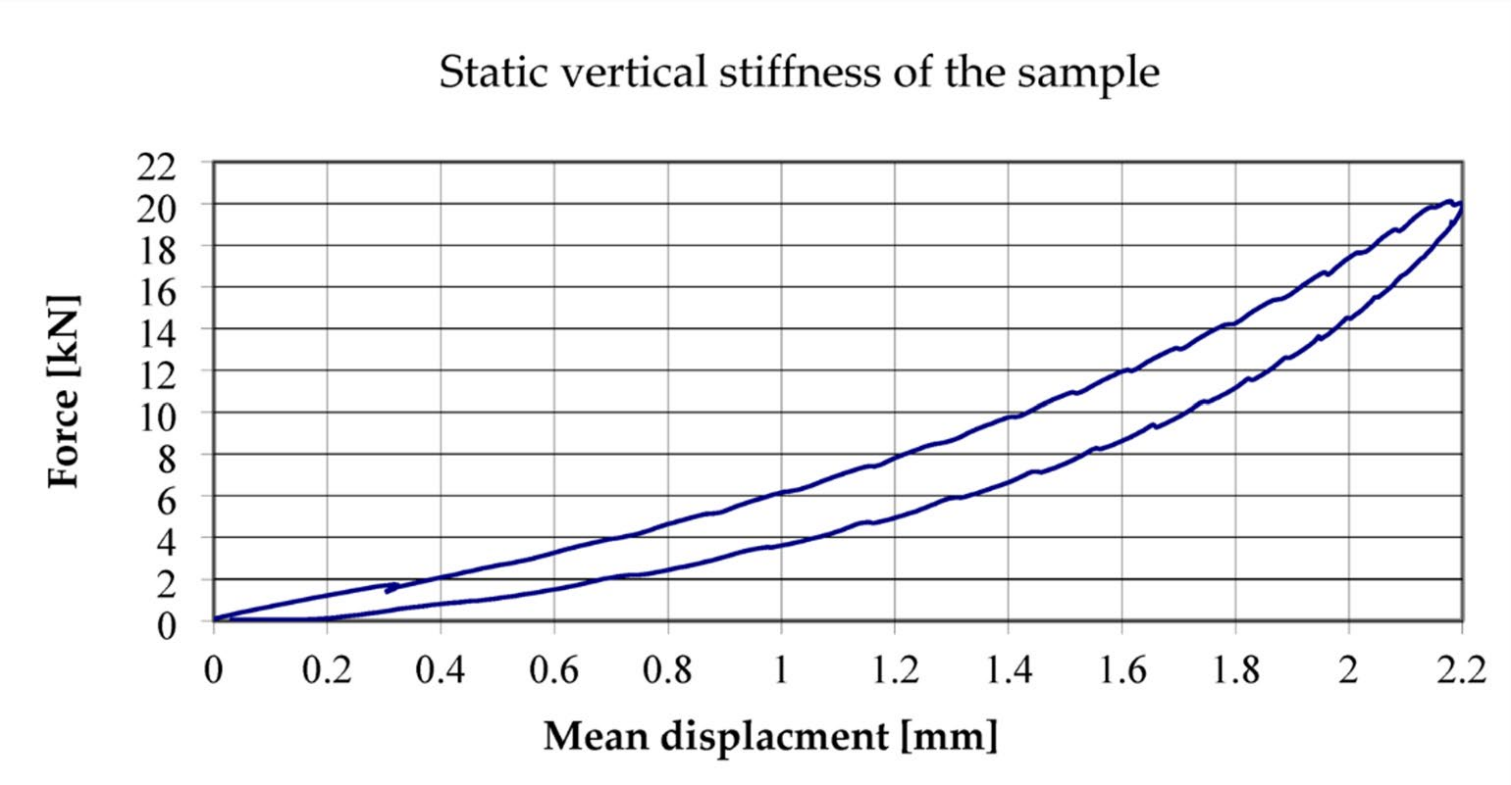
Force–displacement graph for the sample without a concrete block for static stiffness measurement. Own sources.

The static characteristics under a load of up to 20 kN are shown in Fig. 3. A load of 20 kN applied on a surface area of 300 mm x 300 mm corresponds to an approximate load of 150 kN for a prestressed concrete sleeper of type PS-94. For each tested sample, static stiffness was determined using a formula in accordance with the procedure described in the EN 13146-9 + A1:2012 standard^18^:

1 $$\:{K}_{SA}=\frac{{F}_{SA2}-{{F}_{SA}}_{1}}{{d}_{SAmean}}[\mathrm{k}\mathrm{N}/\mathrm{m}\mathrm{m}]$$

where:

*F*_*SA1*_ = 1 kN.

*F*_*SA2*_ = 0,8*F*_*max*;_ *F*_*max*_ =20 kN.

*d*_*SAmean*_ – the displacement along the force application path between *F*_*SA1*_ and *F*_*SA2*_.

The measured static vertical stiffness of the pad was 10.64 kN/mm. Based on this value and the pad dimensions (300 mm × 300 mm), the unit static stiffness was calculated. Here, the unit static stiffness $\:{C}_{stat}$ corresponds to the bedding modulus defined as force per unit area per unit displacement, determined in accordance with EN 16,730. For the pad without confinement, a stiffness of 0.1182 N/mm³ was obtained. This value highlights the inherent material stiffness without interaction effects from ballast confinement.

The same samples that were tested statically were also subjected to dynamic testing, using the same testing equipment. The range of the excitation force was set between 2 and 20 kN, with loading frequencies of 3 Hz, 5 Hz, 10 Hz, and 20 Hz. For each tested sample, dynamic stiffness was determined using a formula in accordance with the procedure described in the EN 13146-9 + A1:2012 standard^19^:

2 $$\:{K}_{LFPf}=\frac{{F}_{LFP2}-{{F}_{LFP}}_{1}}{{d}_{LFP}}\:[\mathrm{k}\mathrm{N}/\mathrm{m}\mathrm{m}]$$

where:

*F*_*LFP1*_ = 1 kN.

*F*_*LFP2*_ = 0,8*F*_*max*;_ *F*_*max*_ =20 kN.

*d*_*LFP*_ – the displacement of assembly in measurement of dynamic low frequency stiffness of assembly for force *F*_*LFP1*_ and *F*_*LP2*_.

**Fig. 4:**
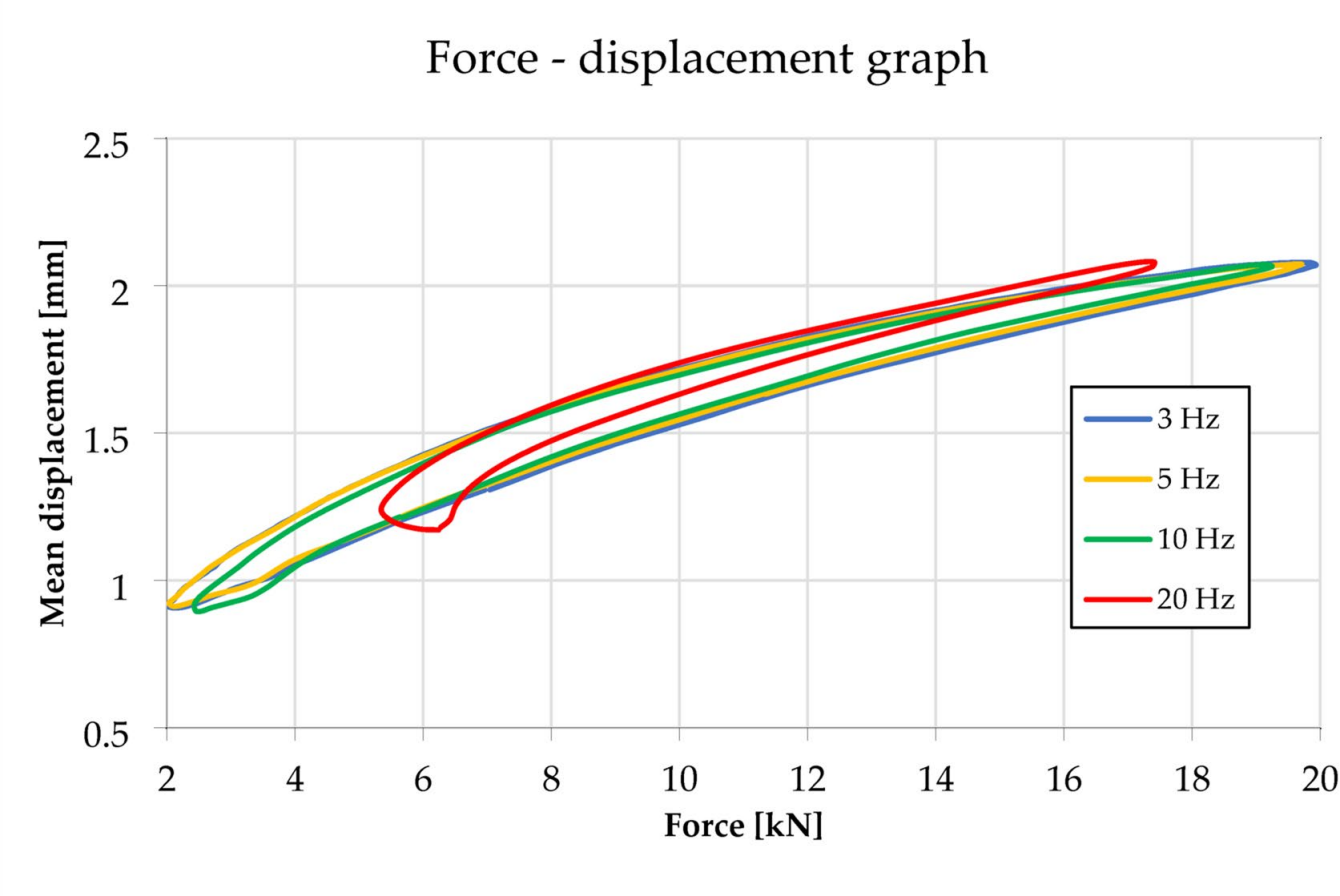
Force–displacement graph for the sample without a concrete block for dynamic stiffness measurement at a frequency of 3 Hz, 5 Hz, 10 Hz, 20 Hz. Own sources.

The dynamic characteristics at 3 Hz, 5 Hz, 10 Hz, and 20 Hz are presented in Figs. 4, respectively. The general value of low-frequency dynamic stiffness was calculated according to standard^19^:

3 $$\:{K}_{LFPmean}=\frac{{F}_{LFP5}+\:{F}_{LFP10}+{F}_{LFP20}\:\:}{3}\:[\mathrm{k}\mathrm{N}/\mathrm{m}\mathrm{m}]$$

Where *K*_*LFP5*_, *K*_*LFP10*_, *K*_*LFP20*_ are measured at 5 Hz, 10 Hz, 20 Hz respectively.

The calculated stiffness values [kN/mm] are summarized in Table 2.

**Table 2:** Dynamic stiffness with different frequency.

Dynamic Stiffness [kN/mm]			
**3 Hz**	**5 Hz**	**10 Hz**	**20 Hz**
15.38	15.46	14.90	12.42

### Test of Under Sleeper Pads with concrete block

#### Test description

In the next stage, the USP samples were bonded to a concrete block representing a prestressed concrete sleeper and tested within the ballast-box setup described previously under harmonic vertical loading (3–10 Hz).

**Fig. 5:**
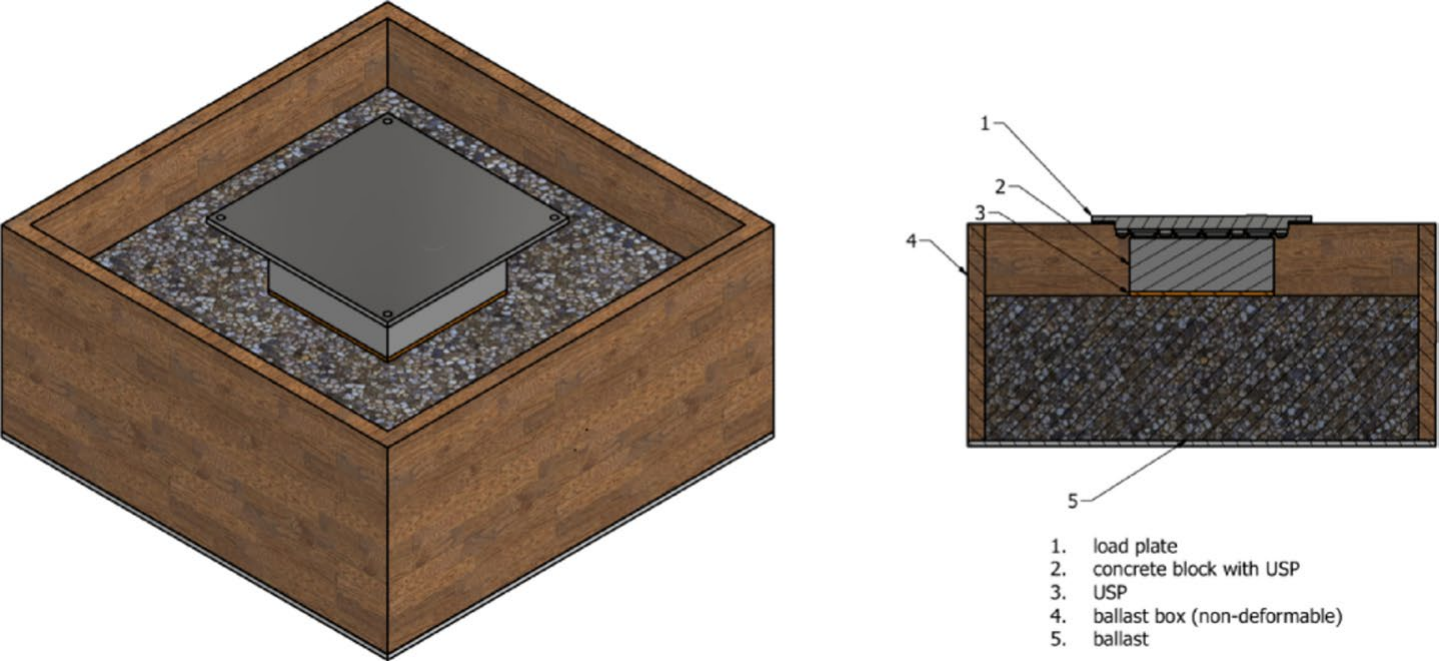
Measurement setup diagram. Own sources.

A schematic top view of the measurement setup is shown in Fig. 5. The block was loaded using a hydraulic actuator. Triaxial accelerometers were installed on the concrete block and on the subgrade surrogate (elastic mat), enabling simultaneous measurement of input and transmitted vibration responses. Vertical displacements of the block were recorded using a displacement transducer.

**Fig. 6:**
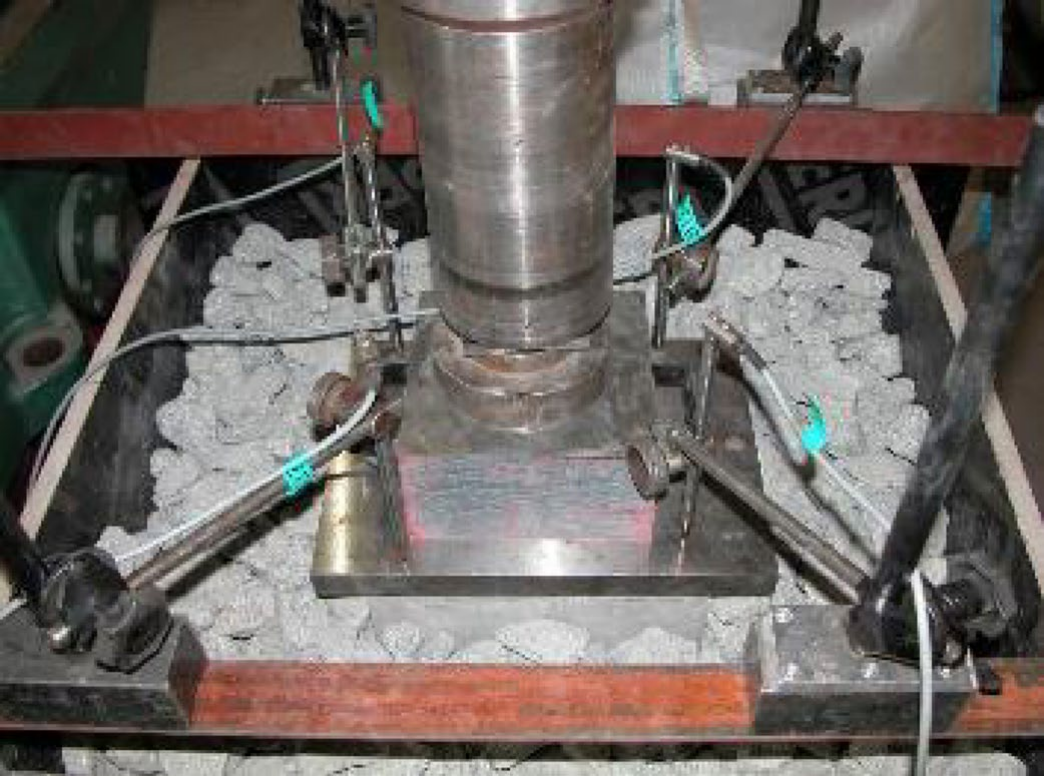
Test stand for static and dynamic stiffness measurements with a concrete block and Under Sleeper pad. Own sources.

Figure 6 shows the test setup with the concrete block and the sinusoidal pad sample placed in a box filled with aggregate. The obtained stiffness values, expressed in [kN/mm], are presented in Table 3. The unit static stiffness of the system, taking into account the dimensions of the pad (300 mm x 300 mm), was 0.0511 N/mm³.

#### Results

**Fig. 7:**
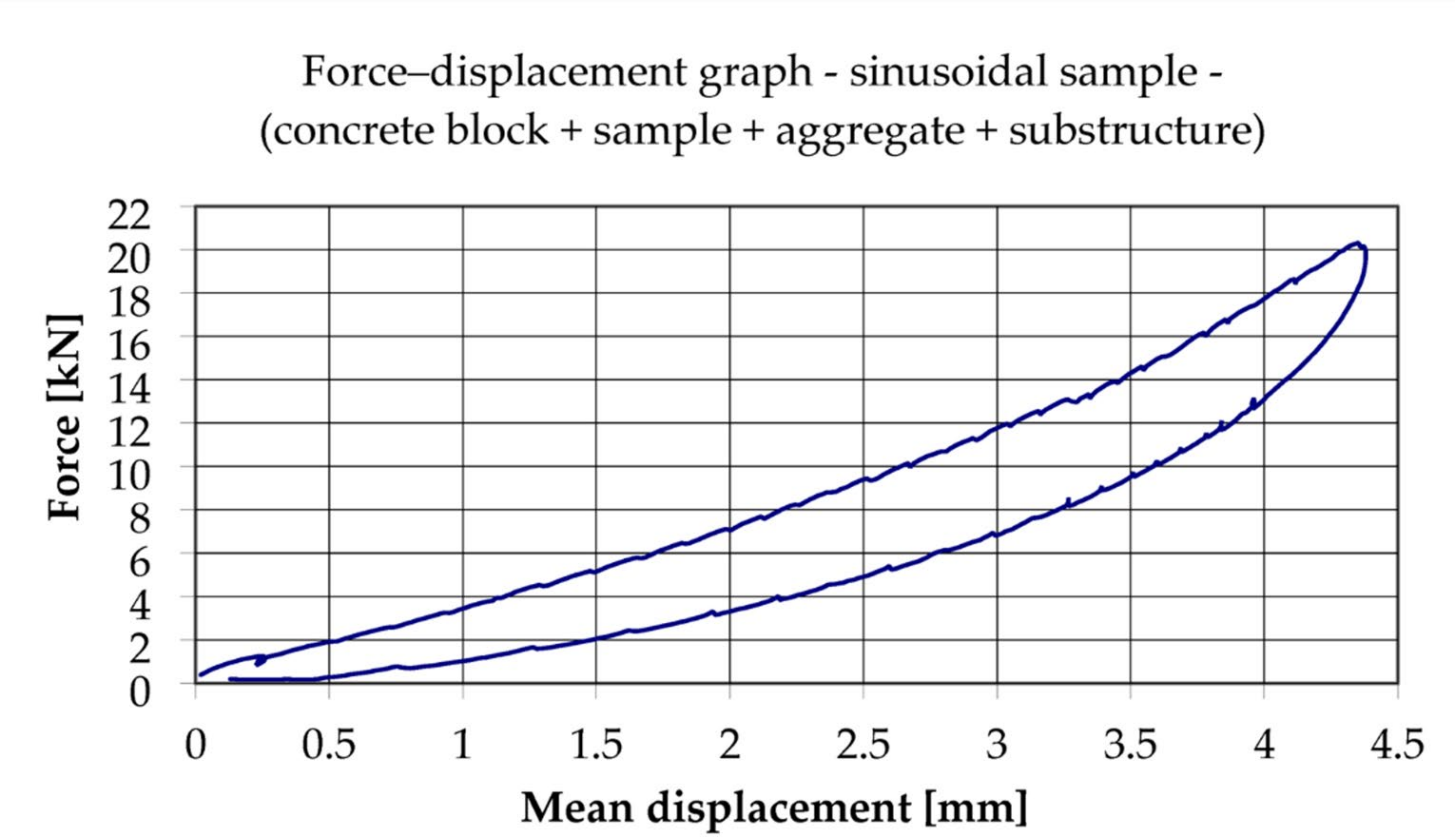
Force – displacement graph for the sample on a concrete block for static stiffness measurement for sample S2. Own sources.

**Fig. 8:**
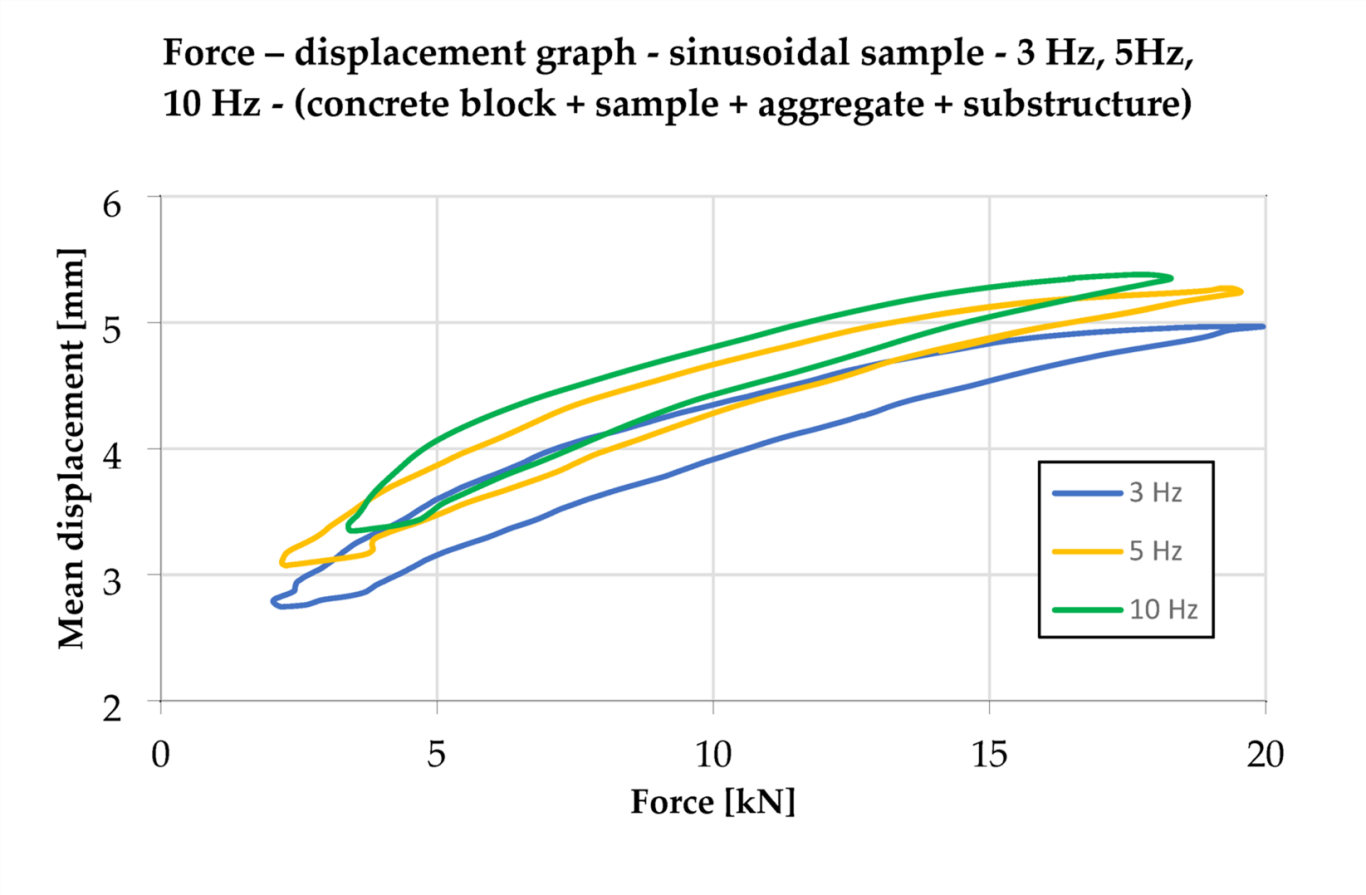
Force – displacement graph for the sample on a concrete block for dynamic stiffness measurement at a frequency of 3 Hz, 5 Hz, 10 Hz for sample S3. Own sources.

Dynamic characteristics were determined at frequencies of 3 Hz, 5 Hz, and 10 Hz. Similarly to the case of the standalone pad, the unit static stiffness was calculated — in this case, it refers to the global stiffness of the system: “concrete block – pad – ballast layer (300 mm) – mat simulating the subgrade”. Figures 7 and 8 present the force – displacement diagrams for the vertical static and dynamic stiffness at frequencies of 3 Hz, 5 Hz, and 10 Hz.

**Table 3:** Static and dynamic stiffness of the block–USP–ballast system.

Dynamic Stiffness [kN/mm]			
**Static Stiffness [kN/mm]**	**3 Hz**	**5 Hz**	**10 Hz**
4.60	7.48	7.71	7.19

The reduction of global system stiffness compared to the standalone pad test confirms the influence of ballast confinement and layered interaction on the effective dynamic response of the assembly.

### Test of Under Sleeper Pads on concrete sleepers

#### Test description

**Fig. 9:**
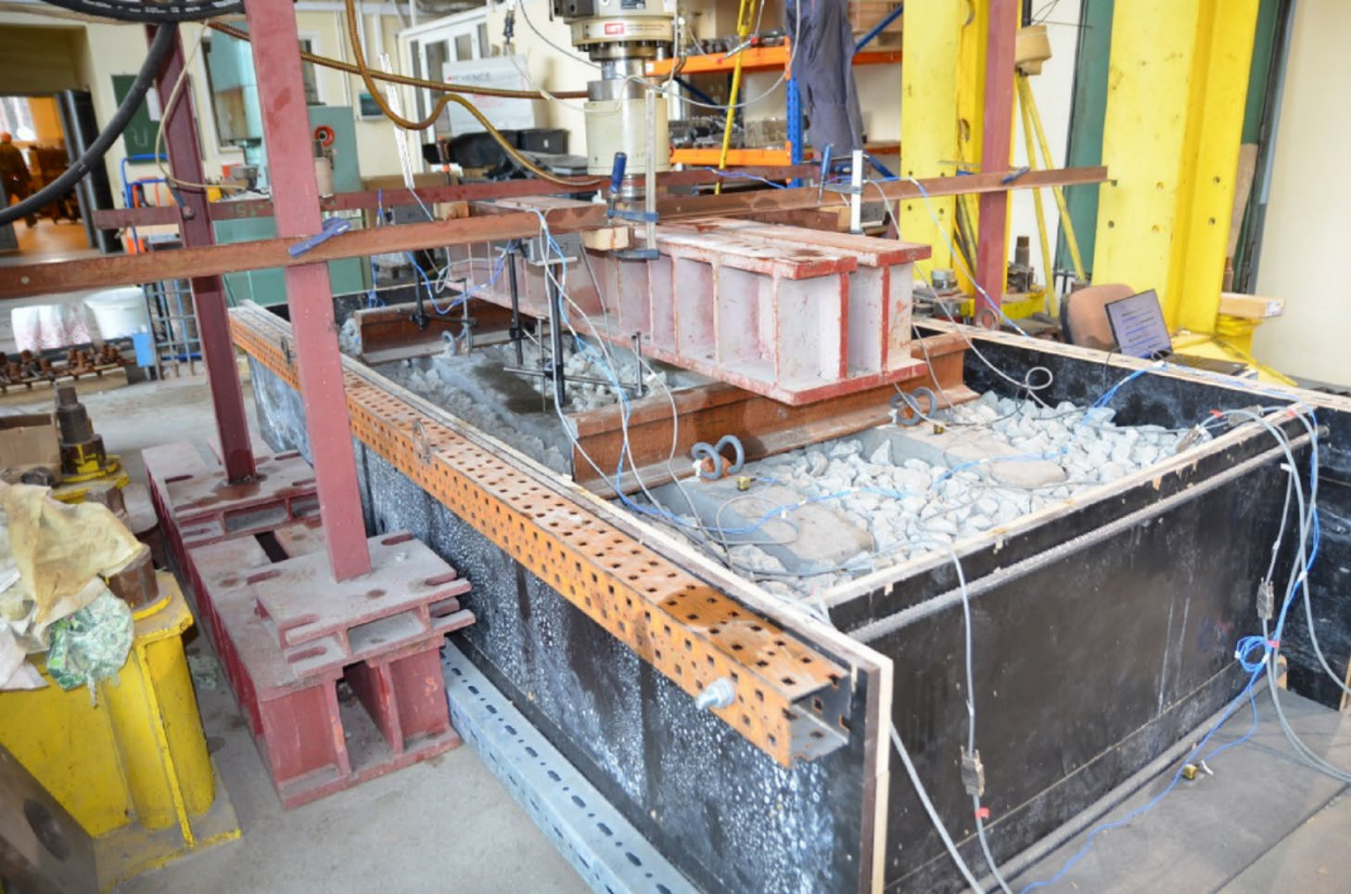
Test setup of sleepers with Under Sleeper pads in a ballast box. Own sources.

Prior to vibration transmissibility evaluation, a fatigue test was conducted on sleeper–USP assemblies installed in a ballast-filled box. The system was subjected to approximately 1.4 million loading cycles at a frequency of 3 Hz. No visible damage, cracking, or detachment of the pads was observed after completion of the loading program (Fig. 9), confirming adequate bonding integrity and material durability under repeated loading.

In the next stage, it was decided to conduct tests to evaluate the effectiveness of vibration reduction in the subgrade using sleeper pads under laboratory conditions.

**Fig. 10:**
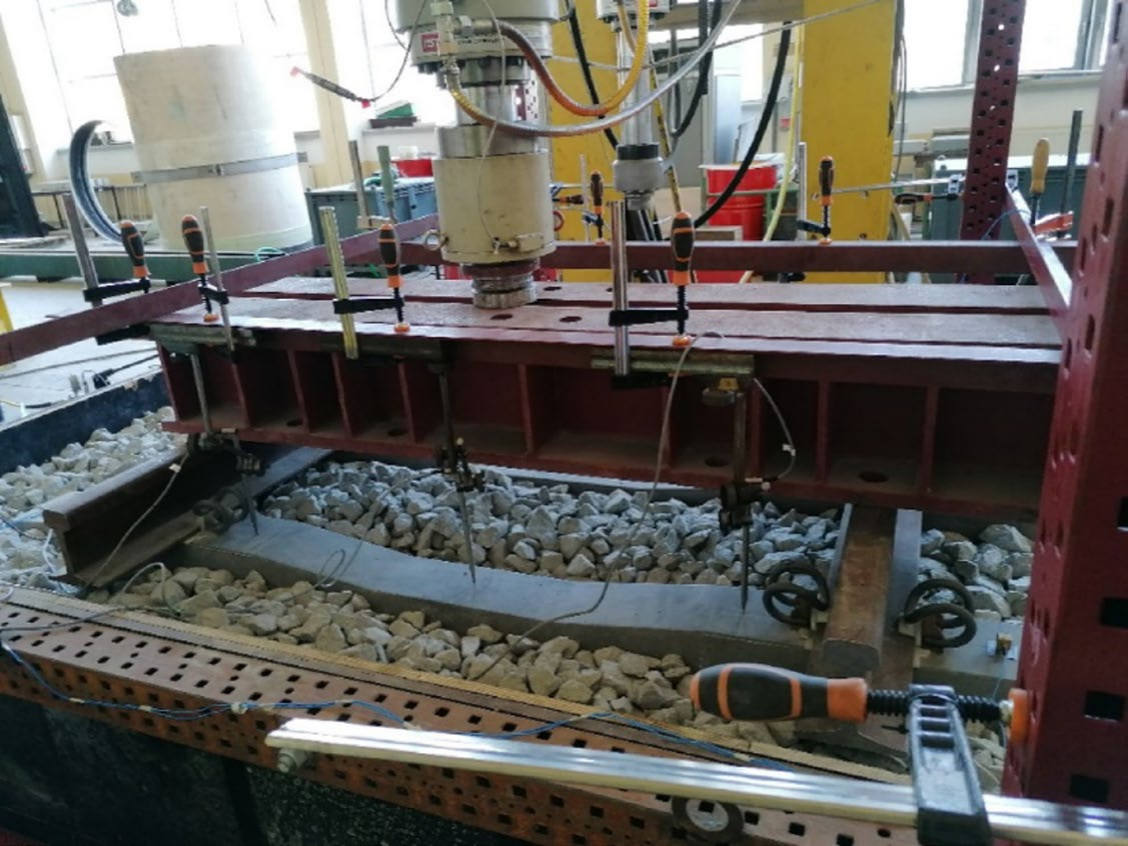
Testing setup for examining the effectiveness of track vibration reduction. Own sources.

**Fig. 11:**
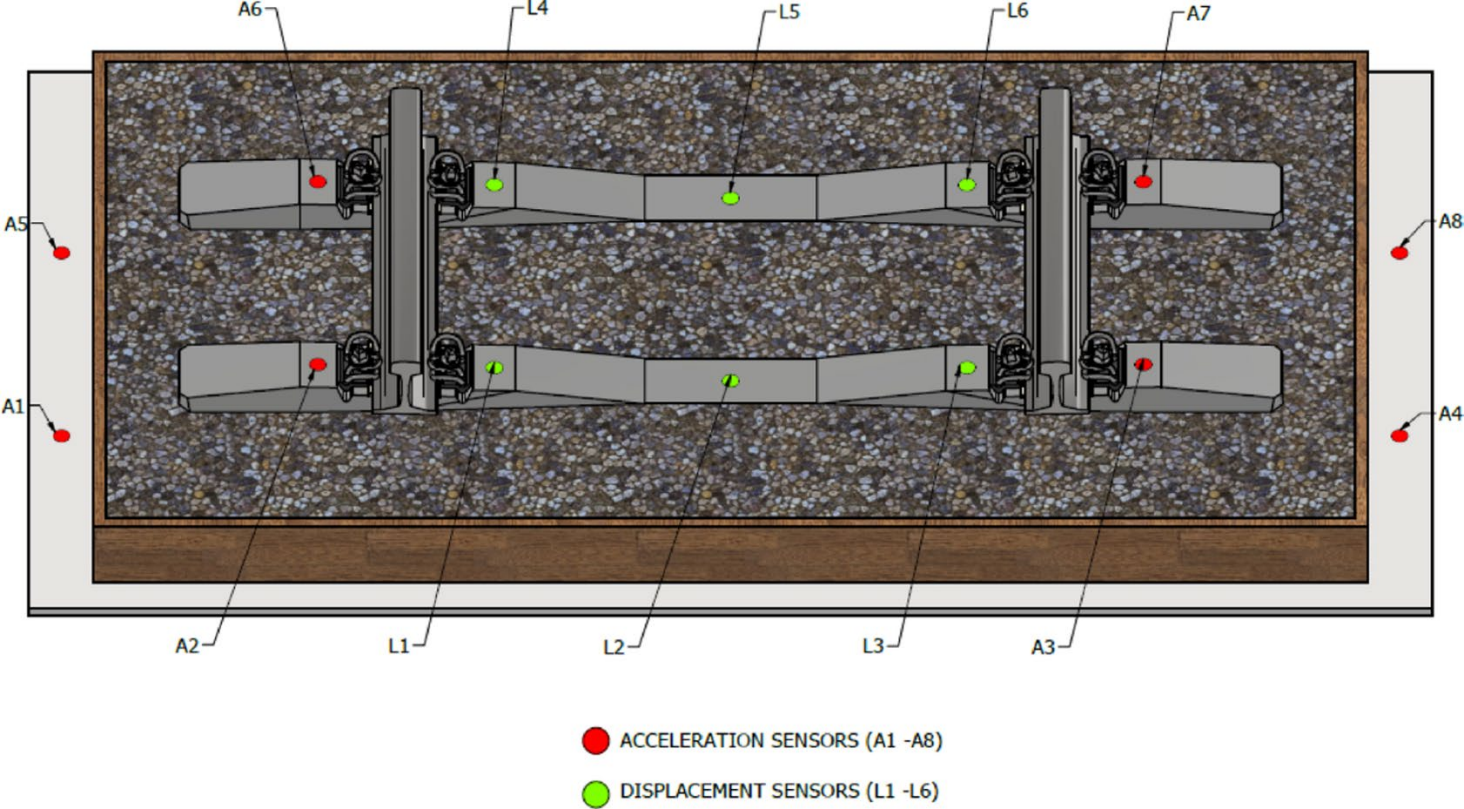
Diagram of sensor placement on the testing setup. Own sources.

A ballast layer with a nominal thickness of 350 mm (under the block) was placed in a box with dimensions of 800 × 800 × 700 mm (Fig. 10). An elastic mat with a thickness of 25 mm was placed under the ballast. This mat served as a laboratory model of the subgrade. Sensors were installed on the concrete block and the subgrade mat simultaneously (Fig. 11). The tests were conducted in two main variants: the first — block without a pad, and the second — block with an Under Sleeper Pad (several types of pads, differing in both stiffness and the shape of the bottom surface — in contact with the ballast). Each measurement (with and without the pad, for each pad type) was conducted in three trials for four forcing frequencies — 3 Hz, 5 Hz, 10 Hz, and 20 Hz. Five types of Under Sleeper Pads with varying static and dynamic stiffnesses were tested in the lab: S1, S2, S3, S4, S5.

#### Results

To quantify vibration isolation performance, dynamic tests were performed using the instrumented ballast-box setup described in Sect.  3.2. Measurements were conducted for two configurations: (i) baseline without a USP and (ii) with a USP installed between the block and ballast. Five USP variants (S1–S5), differing in stiffness and bottom-surface geometry, were tested. Each configuration was evaluated in three repetitions at excitation frequencies of 3, 5, 10, and 20 Hz.

Transmissibility was defined as the ratio of subgrade-to-block responses for RMS acceleration and peak-to-peak displacement.

**Fig. 12:**
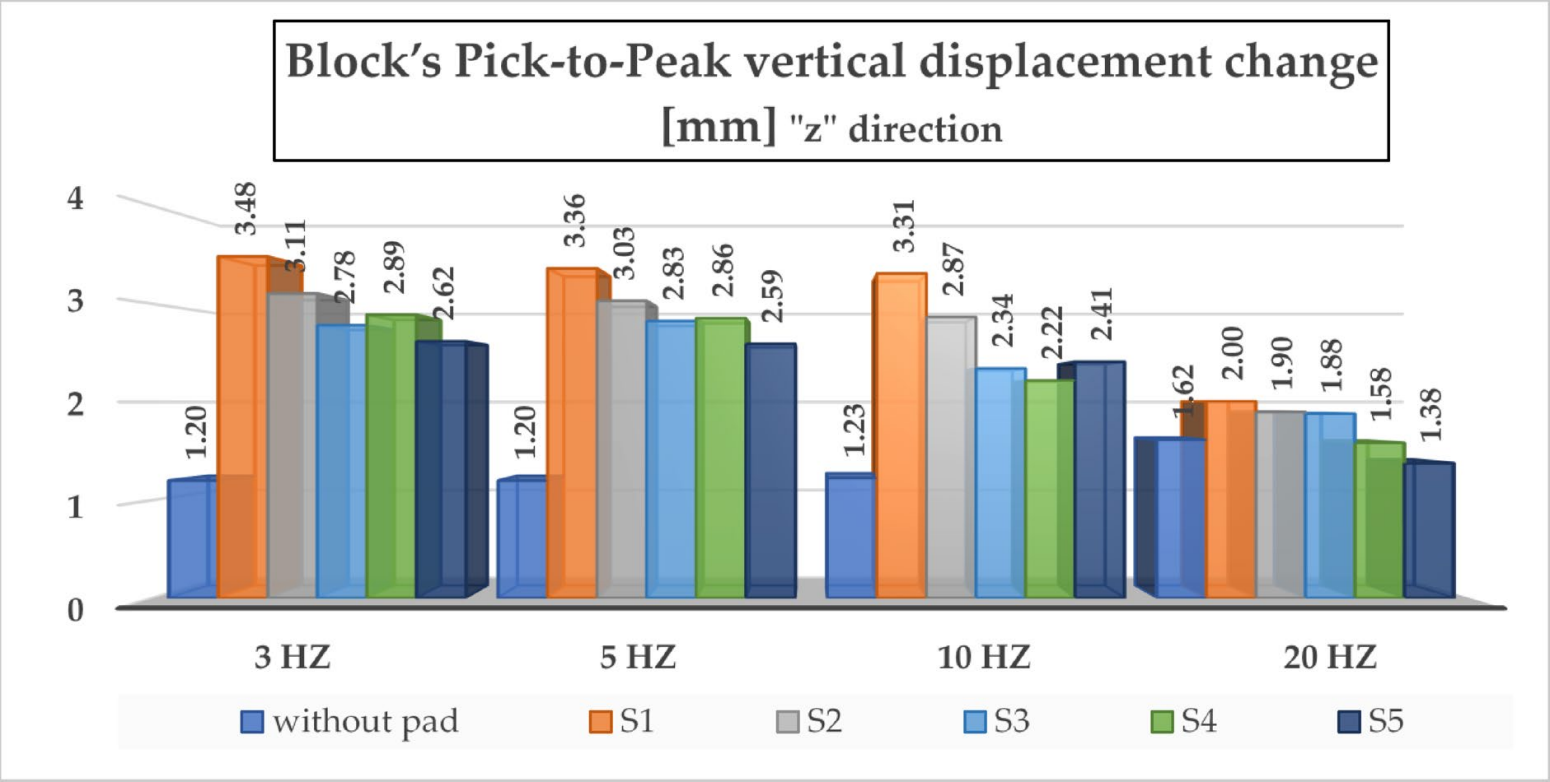
Displacement graph of the concrete block as a function of frequency. Own results.

**Fig. 13:**
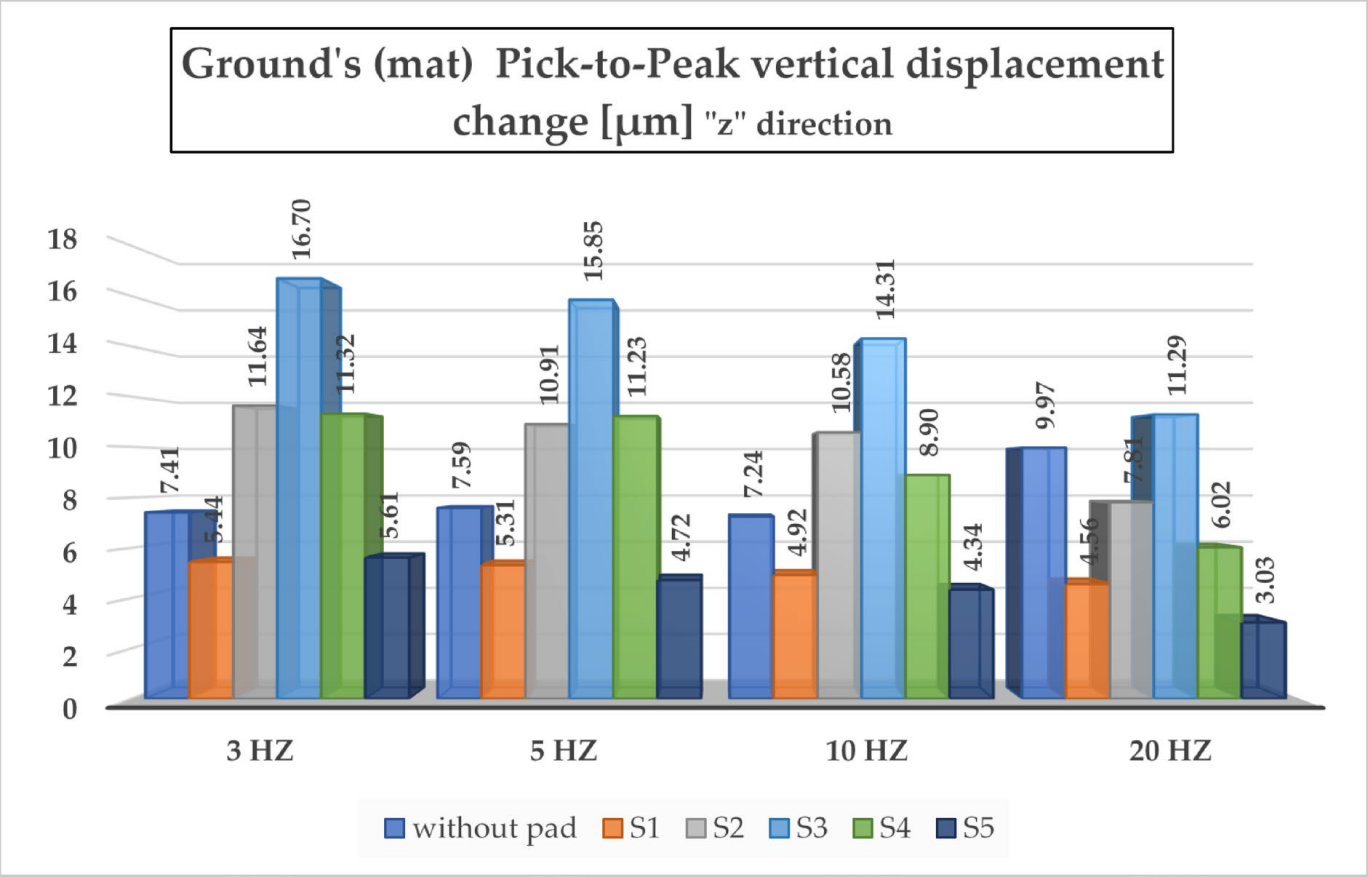
Displacement graph of the mat under the box as a function of frequency. Own results.

Figures 12 and 13 present the absolute displacement amplitudes of the concrete block and the subgrade mat, respectively, as functions of excitation frequency. For all tested frequencies (3, 5, 10 and 20 Hz), the displacement amplitudes measured at the concrete block were consistently higher than those recorded at the subgrade level. This confirms that the Under Sleeper Pad introduces additional compliance at the sleeper–ballast interface, increasing the mobility of the block while attenuating vibration transmission toward the supporting layers.

The observed behavior is consistent with the dynamic response of layered elastic systems. The introduction of a compliant interlayer increases local displacements at the upper element (block), while reducing the propagation of vibration energy into the lower layers. Across all excitation frequencies, the transmissibility values for configurations with USPs were lower than those for the baseline configuration without a pad, confirming the vibration isolation effect.

Acceleration measurements were conducted simultaneously on the concrete block and on the subgrade mat using triaxial accelerometers. For example, at an excitation frequency of 10 Hz, the effective acceleration recorded on the block in the configuration without a pad was approximately 190 cm/s², whereas the corresponding value at the subgrade was about 10 cm/s². After introducing a USP, the effective acceleration measured on the block increased due to enhanced compliance, while the subgrade acceleration decreased by up to 40–50%, depending on pad stiffness. Consequently, the acceleration transmissibility decreased from approximately 0.05 in the no-pad configuration to below 0.02 for the most compliant pad variants.

A similar trend was observed for displacement-based estimators. The peak-to-peak displacements of the block increased when a USP was installed, whereas the corresponding displacements of the subgrade decreased significantly compared to the baseline configuration. The consistent condition $\:{u}_{block}>{u}_{subgrade}$ across the entire frequency range confirms that the laboratory model did not exhibit local amplification at the subgrade level within the investigated excitation range and that the observed isolation effect is physically meaningful.

In certain configurations, a moderate decrease in displacement amplitude with increasing excitation frequency was observed. This trend can be attributed to the frequency-dependent stiffness characteristics of the ballast–mat system. As excitation frequency increases within the investigated range, the effective dynamic stiffness of the layered system increases relative to the applied harmonic load, resulting in reduced displacement amplitudes. Such behavior is typical for multi-layer elastic systems operating below resonance.

Overall, the experimental results demonstrate that the application of Under Sleeper Pads significantly improves the vibration isolation performance of the track model. The effectiveness of isolation depends strongly on pad stiffness and bottom-surface geometry. Based on the quantitative comparison of transmissibility values across all tested frequencies, the pad variant providing the lowest acceleration and displacement transmissibility was selected for further in-situ verification under operational railway conditions.

This study has several limitations. The experiments were conducted under reduced-scale laboratory conditions with harmonic excitation and simplified boundary conditions. Therefore, the results may not fully represent real in-track conditions under variable train loading. In addition, long-term performance and environmental effects were not assessed. Full-scale field validation is required to confirm the applicability of the findings.

## Conclusions

The conducted laboratory investigations confirmed that the application of Under Sleeper Pads (USPs) significantly reduces vibration transmission from the sleeper to the track substructure. The experimental results consistently demonstrated a characteristic trade-off: the introduction of an elastic interlayer increases the dynamic response of the sleeper substitute while attenuating vibration levels measured at the subgrade surrogate.

For all tested excitation frequencies (3–20 Hz), the condition $\:{u}_{block}>{u}_{subgrade}$ was satisfied, confirming that the investigated system operated below resonance and that no local amplification occurred at the subgrade level. Both acceleration- and displacement-based transmissibility functions showed clear reduction in vibration transfer when USPs were applied. The degree of isolation depended strongly on pad stiffness and bottom-surface geometry, with more compliant and corrugated variants providing the lowest transmissibility values.

The results highlight the importance of selecting pad mechanical properties in accordance with the expected operational and structural conditions of the railway line. While softer pads offer improved vibration isolation, they simultaneously increase sleeper mobility, which must be considered in track design and long-term performance assessment.

It should be noted that the present study was conducted under reduced-scale laboratory conditions with harmonic excitation and simplified boundary constraints. Although the experimental model was designed to replicate key mechanical interactions within the ballast–sleeper–subgrade system, full-scale field validation is necessary to confirm long-term performance under real traffic loading.

Based on the quantitative comparison of transmissibility indicators, one optimized USP variant, characterized by a unit static stiffness of approximately 0.12 N/mm³ and a corrugated contact surface, was selected for planned in-track verification on the PKP PLK railway network.

## Data Availability

The datasets used and/or analysed during the current study are available from the corresponding author on reasonable request.
